# Successful Treatment of Obstructive Pneumonia with Hemoptysis due to Non-Absorbable Suture-Related Granulation

**DOI:** 10.70352/scrj.cr.25-0236

**Published:** 2025-07-02

**Authors:** Yukitaka Sato, Hironori Ishibashi, Ryota Ishizawa, Ayaka Asakawa, Kenichi Okubo

**Affiliations:** Department of Thoracic Surgery, Institute of Science Tokyo, Bunkyo-ku, Tokyo, Japan

**Keywords:** granulation tissue, foreign material, non-absorbable suture, hemoptysis, endobronchial stenting

## Abstract

**INTRODUCTION:**

Non-absorbable sutures or Teflon pledgets (model number: 00801741041341, Bard, Franklin Lakes, NJ, USA) are sometimes used for protection of the bronchial stump to prevent bronchial fistula. However, there have been reports of foreign body-related bronchial granulation in the distant phase. Treatment of this rare complication is challenging, and there are no reports in the literature of cases that ultimately underwent curative surgical excision.

**CASE PRESENTATION:**

A 63-year-old man underwent a right lower lobectomy with ND2a-2 for typical pulmonary carcinoid 20 years ago. Twelve years after the operation, the right intermediate bronchus gradually became obstructed with granulation tissue from the right lower bronchial stump. Therefore, we eliminated the obstruction and placed a 2-cm Dumon stent (model number: 20300BZY00250000, Novatech SA, La Ciotat, France) in the intermediate bronchus. However, the inner lumen of the stent gradually became filled with the granulation tissue, and 6 years after the stenting, the patient was referred to the hospital owing to massive hemoptysis and obstructive pneumonia. Although transcatheter bronchial arterial embolization was performed for a pseudoaneurysm, blood-tinged sputum remained present, and aspiration pneumonia had spread to the right upper lobe. Bronchoscopy showed that a non-absorbable suture, which was used for the protection of the bronchial stump 20 years ago, was buried in the obstructive tissue. After antibiotic treatment for the pneumonia, we performed a right middle lobectomy as well as the removal of the stent and the threads as a curative treatment.

**CONCLUSIONS:**

Non-absorbable suture sometimes causes granulation tissue in the distant phase, and absorbable sutures are preferable for the bronchial stump. For the treatment, complete excision at an appropriate time is required based on the severity of the symptoms.

## Abbreviations

BAE
: bronchial arterial embolization
CT
: computed tomography

## INTRODUCTION

Bronchial fistula after lung resection still remains a serious complication with a high mortality rate. To prevent this complication, non-absorbable sutures or Teflon pledgets (model number: 00801741041341, Bard, Franklin Lakes, NJ, USA) are sometimes used for protection of the bronchial stump.^1)^ However, there have been reports that these foreign materials can become exposed in the bronchus and cause granulation tissue formation, which obstructs the airways.^2,3)^

Here, we report a rare case of obstructive pneumonia with hemoptysis caused by non-absorbable suture-associated granulation after right lower lobectomy for a carcinoid tumor.

## CASE PRESENTATION

**Fig. 1:**
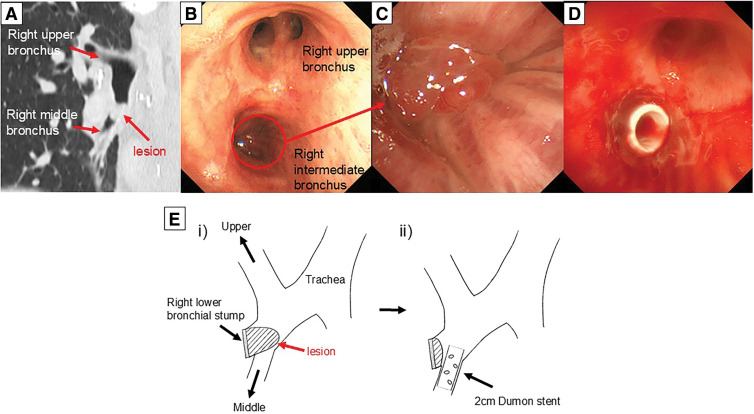
(**A**–**C**) CT scan and bronchoscopy revealed an endobronchial polyp obstructing the right intermediate bronchus. (**D**) A 2-cm Dumon stent (model number: 20300BZY00250000, Novatech SA, La Ciotat, France) was placed from the entrance of the intermediate bronchus to the right middle bronchus. (**E**(**i**, **ii**)) Schematic diagrams of before and after stenting. CT, computed tomography

A 63-year-old man underwent a right lower lobectomy with ND2a-2 for typical bronchial carcinoid 20 years ago at the previous hospital. Twelve years after the operation, a CT scan revealed an endobronchial polyp arising from the bronchial stump, which was diagnosed as granulation tissue by bronchoscopic biopsy. The polyp gradually obstructed the right intermediate bronchus, and the patient was referred to our hospital (**Fig. 1A**–**1C** and **Ei**). We placed a 2-cm Dumon stent (model number: 20300BZY00250000, Novatech SA, La Ciotat, France) from the entrance of the intermediate bronchus to the right middle bronchus after excision of the granulation using argon plasma coagulation (**Fig. 1D** and **Eii**).

Annual follow-up CT scans showed that the inner lumen of the stent gradually became filled with the granulation tissue; however, since there were no symptoms such as fever or hemoptysis, we decided to leave the stent in place.

**Fig. 2:**
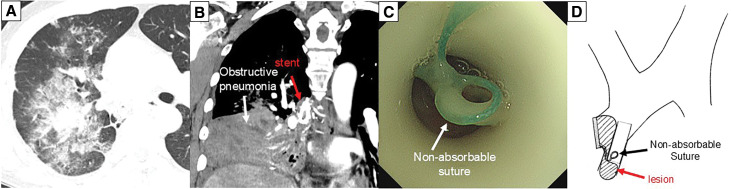
(**A**, **B**) CT scan revealed right upper lobe aspiration pneumonia and complete middle lobe obstructive pneumonia. (**C)** Bronchoscopy revealed a non-absorbable suture in the stent. (**D**) Schematic representation of stent re-obstruction. CT, computed tomography

Six years after the stenting, he was transported to the previous hospital for massive hemoptysis. A CT scan revealed right upper lobe aspiration pneumonia and complete middle lobe obstructive pneumonia caused by the rupture of a bronchial arterial pseudoaneurysm (**Fig. 2A** and **2B**). After emergent BAE, the patient was transferred to our hospital for emergency surgery to prevent fatal hemoptysis.

**Fig. 3:**
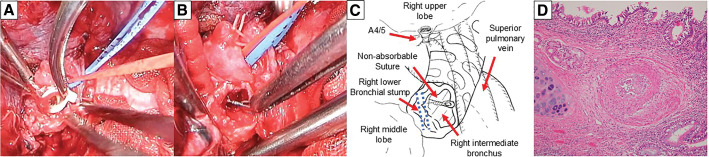
(**A**, **B**) After the stent was removed, non-absorbable sutures were identified in the proximal part of the intermediate bronchus. (**C**) Schematic representation of the intraoperative findings. (**D**) Microscopically, the lesion was not malignant and was diagnosed as granulation tissue with fibrosis.

The patient was receiving oxygen via a nasal cannula at 3 L/min, and bronchoscopy revealed no active airway bleeding, although a non-absorbable suture, which was used for the protection of the bronchial stump 20 years ago, was found buried in the granulation tissue (**Fig. 2C** and **2D**). He received hemostatic agents and antibiotics for 2 weeks, and the right upper lobe aspiration pneumonia improved. We performed a posterolateral open right middle lobectomy as well as the removal of the stent and the threads as a curative treatment. Adhesions of the thoracic wall and vessels around the hilum were severe. After cutting the distal part of the middle bronchus, the stent and non-absorbable sutures were identified in the intermediate bronchus (**Fig. 3A**–**3C**). The bronchial stump was sutured using absorbable 4-0 polydioxanone sutures and covered with free pericardial fat. Microscopically, the lesion showed no malignancy and was diagnosed as granulation tissue (**Fig. 3D**).

The patient was discharged on POD 12 and was followed uneventfully for 2 years.

## DISCUSSION

Foreign body-associated bronchial granulation is a rare postoperative complication in the thoracic field, and its treatment is challenging. Some reports indicate that bronchoscopic management, including endobronchial stenting, can be effective for postoperative granulation.^2,3)^ However, granulation tissue tends to recur unless the underlying cause—the presence of a foreign body—is eliminated, and these interventional methods themselves carry a potential risk for inducing granulation.^4)^ According to Roodenburg et al.,^5)^ granulation tissue may also develop in response to lung-implantable devices due to procedure-related tissue injury and device-related factors. In this case, the granulation tissue was caused by non-absorbable sutures, and interventional stenting proved ineffective. A follow-up CT scan revealed that the inner lumen of the stent was gradually obstructed again, and the long-term placement of the stent may have further contributed to the onset of hemoptysis. However, the patient remained asymptomatic until massive hemoptysis occurred. We decided to leave the stent in place, considering the risks of complete re-obstruction of the right intermediate bronchus after stent removal, as well as the risk of bleeding associated with bronchoscopic procedures. In treatment-resistant bronchial obstruction, foreign body-associated granulation should be considered, and curative surgical excision might be required.

Hemoptysis is one of the life-threatening symptoms of respiratory diseases, and adequate treatments are therefore required. In recent years, with the widespread adoption of BAE, Andréjak et al.^6)^ reported that performing BAE prior to surgery can help optimize the patient's condition and avoid emergent surgery, ultimately improving patient outcomes. A meta-analysis showed a clinical success rate of BAE equal to 92.46%. However, as the recurrence rates after this procedure are 21.46%, many cases still require curative surgical treatment.^7)^ In this case, chronic obstructive pneumonia led to massive hemoptysis. Although emergent surgery, including pneumonectomy, was first considered because the aspiration pneumonia was severe even after BAE, the upper lobe was ultimately preserved after adequate multidisciplinary treatment.

## CONCLUSIONS

We present a successful case of massive hemoptysis from obstructive pneumonia caused by non-absorbable suture-related granulation 20 years after surgery.

Non-absorbable sutures sometimes cause bronchial granulation tissue in the distant period, and curative surgical excision will be required based on the severity of the symptoms.

## ACKNOWLEDGMENTS

We would like to thank Editage (www.editage.com) for English language editing.

## DECLARATIONS

### Funding

None declared.

### Authors’ contributions

YS and HI conceived the idea of this report.

YS drafted the original manuscript.

HI and KO supervised the writing of the manuscript.

YS, HI, AA, and RI performed the surgery and perioperative management of the patient.

All authors are responsible for the manuscript and approved the final version.

### Availability of data and materials

The data underlying this article will be shared upon reasonable request to the corresponding author.

### Ethics approval and consent to participate

All procedures followed were in accordance with the ethical standards of the responsible committee on human experimentation (institutional and national) and the 1964 Declaration of Helsinki and its later versions. This study was approved by the Institutional Review Board of the Institute of Science Tokyo Hospital (certificate number: M2017-326).

### Consent for publication

The patient provided written consent for publication.

### Competing interests

None declared.
